# Challenges of Prevention for a Sustainable Personalized Medicine

**DOI:** 10.3390/jpm11040311

**Published:** 2021-04-16

**Authors:** Roberta Pastorino, Claudia Loreti, Silvia Giovannini, Walter Ricciardi, Luca Padua, Stefania Boccia

**Affiliations:** 1 Department of Woman and Child Health and Public Health—Public Health Area, Fondazione Policlinico Universitario A. Gemelli IRCCS, 00168 Rome, Italy; roberta.pastorino@policlinicogemelli.it (R.P.); stefania.boccia@unicatt.it (S.B.); 2 Dipartimento di Scienze dell’Invecchiamento, Neurologiche, Ortopediche e della Testa-Collo, Fondazione Policlinico Universitario A. Gemelli IRCCS, 00168 Rome, Italy; silvia_giovannini@yahoo.it (S.G.); luca.padua@unicatt.it (L.P.); 3 Sezione di Igiene, Dipartimento Universitario di Scienze della Vita e Sanità Pubblica, Università Cattolica del Sacro Cuore, 00168 Rome, Italy; walter.ricciardi@unicatt.it; 4 Dipartimento di Scienze Geriatriche e Ortopediche, Università Cattolica del Sacro Cuore, 00168 Rome, Italy

**Keywords:** personalized medicine, prevention, polygenic risk score, rehabilomics

## Abstract

The development and implementation of the approaches of personalized medicine for disease prevention are still at infancy, although preventive activities in healthcare represent a key pillar to guarantee health system sustainability. There is an increasing interest in finding informative markers that indicate the disease risk before the manifestation of the disease (primary prevention) or for early disease detection (secondary prevention). Recently, the systematic collection and study of clinical phenotypes and biomarkers consented to the advance of Rehabilomics in tertiary prevention. It consents to identify relevant molecular and physiological factors that can be linked to plasticity, treatment response, and natural recovery. Implementation of these approaches would open avenues to identify people at high risk and enable new preventive lifestyle interventions or early treatments targeted to their individual genomic profile, personalizing prevention and rehabilitation. The integration of personalized medicine into prevention may benefit citizens, patients, healthcare professionals, healthcare authorities, and industry, and ultimately will seek to contribute to better health and quality of life for Europe’s citizens.

## 1. Introduction

The personalization and sustainability of health systems is on top of European Union and national governments’ agendas. In the past decade, a variety of challenges including population ageing, increasing prevalence of chronic conditions and short-term costs of new therapies led to substantial rise in health expenditures, putting sustainability of European health systems at risk. In 2019, 20.3% of the EU-27 population was aged 65 and over, and the share of people aged 80 years or above in the EU-27’s population is projected to increase by 2.5 fold between 2019 and 2100 (from 5.8% to 14.6%) [1]. As such, all EU Member States face growing pressures to ensure the quality, effectiveness, and efficiency of their health systems within the capacity of their public budgets.

Moreover, the challenges posed by the Coronavirus pandemic (COVID-19) highlighted the urgent need to radically transform our health care services in order to make the health care systems resilient and sustainable overall.

As highlighted by the European Steering Group on Sustainable Healthcare and reaffirmed in the State of the Health of EU of the Companion report, tangible innovative actions are needed to implement a sustainable healthcare, “from acute to chronic care, from treatment of established disease to prevention and early diagnosis, from hospital dependency to integrated care across all levels of health systems, as well as from cost and volume to value and outcome” [2,3].

Value can be increased by making personalized medicine (PM) an integral part of the approach to population health management and by the application of appropriate, timely, and well-coordinated health policies that slow the rate of health decline associated with ageing and thus the amount of health care services required. One third of the EU adult population, in fact, is currently affected by at least one chronic disease that contributes to 75% of the mortality, and on average 18 years of the last period of life are spent with at least one disability [4].

While approaches of PM are already being implemented for disease diagnosis and treatment, development and implementation of such approaches for prevention are still at infancy. In 2019, the Personalized PREvention of Chronic DIseases (PRECeDI) consortium published a set of recommendations for policymakers, scientist and industry on how to integrate PM into chronic disease prevention [4]. Several personalized applications can have the potential for more effective prevention of chronic diseases postponing the onset of disabilities and reducing healthcare costs [5]. Over the last twenty years, the incredible progress in genotyping technology, together with the reduction in costs of genome sequencing and the advent of digital technologies in healthcare, including wearable devices and mHealth, has initiated a third revolution in medicine. In this context, there is an increasing interest in finding informative markers that indicate the disease risk before the manifestation of the disease (primary prevention) or for early disease detection (secondary prevention).

However, to ensure effective and efficient development, change in the healthcare systems is required. These will include not only health services reorganizations, but also citizen engagement, health care professional’s education, and the broader social and legal aspects.

## 2. Personalized Medicine in Primary and Secondary Prevention

Current evidence on chronic disease prevention suggests that interventions targeted at high-risk individuals represents the best way forward [6]. Primary prevention aims to prevent disease before it ever occurs [7]. This is done by intervening before health effects occur, through measures such as vaccinations, altering risky behaviors (unhealthy or unsafe behaviors, poor eating habits, tobacco use), and banning substances known to be associated with a disease or health conditions. The focus of secondary prevention, instead, is to reduce the impact of a disease that has already occurred, making it possible to prevent the worsening of the disease and the emergence of symptoms [8]. This is done by detecting (through screening or diagnostic testing) and treating the disease as soon as possible to halt or slow its progress, encouraging personal strategies to prevent recurrence, and implementing programs to prevent long-term problems.

Nowadays, there is much attention and excitement in the global healthcare environment on the potential of omic information (e.g., genomics, proteomics, metabolomics, radiomics) to support the delivery of PM in healthcare systems. In particular, the potential of genomics to personalize and thereby improve diagnosis, treatment, and prognosis of individuals has long been recognized, but so far evidence of the opportunity for genomics to drive prevention remains limited [4,9].

However, recent advances in research on polygenic risk scores (PRS), defined as a quantitative assessment of the risk of a specific condition based on the collective influence of a number of genetic variants, have created new interest about the use of genetic information in prevention of common diseases and its application to risk stratification [10].

PRS is calculated combining weighted genotypes for risk alleles into a single, integrated measure of an individual’s genetic predisposition to a specific phenotypic profile. PRS are not designed to reveal the complexity of molecular susceptibility mechanisms, but they are highly amenable to phenotypic prediction.

A long-lasting open question was if common genetic variants used to build PRSs were able to predict risk of developing complex diseases with enough power to be used in a clinical setting [10]. The lack of large enough dataset able to allow an unbiased PRS validation, hampered this question to be answered for many years. The institution of new cohorts and biobanks, as the UK Biobank [11], allowed a robust validation of predictive models for complex disease based on PRS. Validation was achieved thanks to a huge number of samples and the possibility to analyze environmental factors, which may be important determinants of risk potentially modifiable through changes in lifestyle or proper interventions (primary and secondary prevention).

Stratification of individuals according to their risk of disease based on PRS can improve prevention strategies like cancer screening programs that fall into secondary preventive interventions. For example, the risk distribution of breast cancer for the lowest vs. highest percentiles varied from a few percent to over 30%. Chatterjee et al. showed how PRSs could help to determine the age at which the risk of developing breast cancer reaches a threshold of 2.4%, the average population 10-year risk at the currently recommended starting age for mammographic screening in the UK [12]. According to the PRS calculated in the study, “women in the top 10% of genetic risk would reach this risk threshold (2.4%) in their early 30s, whereas women in the bottom 10% of the polygenic risk would remain below this threshold throughout their lifetime”, suggesting that they might not need to participate at all to the breast cancer screening program. Thus, information on genetic risk, in addition to family history, is more effective than an age-based criterion in guiding decision making on when mammographic screening should be started [12].

**Figure 1:**
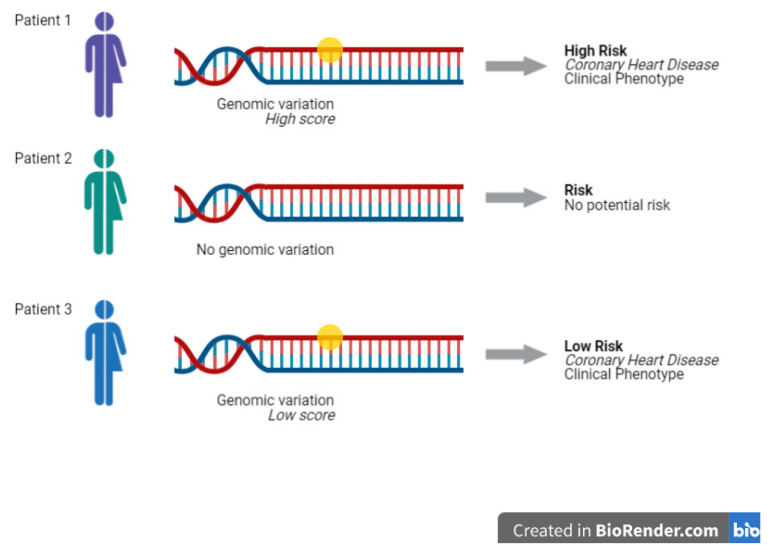
Polygenic Risk Scores test can predict the risk of developing a disease according to the score of the genomic variation investigated. Image created with BioRender.com (accessed on 1 March 2021).

Similar examples can be done for coronary heart disease (CHD) (Figure 1) and colorectal cancer [13,14,15,16]. For instance, several studies illustrates the utility of PRSs for primary and secondary prevention of CHD by reanalysis of data from randomized trials of statin treatment showing that statin intake reduces the absolute risk of CHD to a greater extent in individuals at higher versus lower polygenic risk [13]. Interventions in these high-risk individuals could be compliant with medication, but also close monitoring and lifestyle adaptation.

The relevance of the topic in the field of cancer is also demonstrated by the fact that the latest report of the Mission Board for Cancer lists prevention as one of the three areas of focus, and one of the 13 recommendations concerns the development of a Europe-wide research programme to identify PRS for cancer prevention. In addition, the research programme should promote the clinical validation of PRS, educational activities on the clinical relevance of PRS in all citizens whatever their age, and stimulate public debate on their use and control [17].

## 3. The Challenge of Personalized Medicine in Tertiary Prevention

Tertiary prevention (TP) aims to improve Quality of Life (QoL) of patients decreasing disability, limiting or delaying complications and, where is possible, restoring function. TP aims to reduce the impact of the disease and its complications by treating the disease and providing rehabilitation [17,18]. Rehabilitation plays a central role in several disease recovery, in particular in fragile patients and in multidimensional complex pathologies [19,20,21,22], and it is constantly evolving. Recently, so-called “tailored rehabilitation” is a critical topic because nowadays a very large number of different approaches are available: besides conventional therapy, technological treatments have become accessible [23,24]. To achieve personalization in TP, it is crucial to maximize its effectiveness and responsiveness by individually orienting and tailoring treatments according to the specific needs and characteristics of each single person [25,26,27,28,29].

### 3.1. The Example of Severe Brain Injuries

Despite many patients presenting similar clinical manifestation, outcome of rehabilitation may differ for patients with complex multidimensional injuries such as severe brain injuries [29,30]. Severe brain injuries are a major cause of mortality and disability, their pathophysiology and recovery are both complex and varied. Because many severe traumas occur unpredictably, personalization towards the “-omics” is hard to be achieved in primary and secondary preventions. Due to the nature of severe brain traumas, rehabilitation does not lend itself to a singular “protocolized” and standardized plan of therapy. Yet, by necessity, it operates as a functional model of personalized care [30]. It is therefore crucial finding the “right” treatment for each patient to pursue TP main goal: improving QoL and decreasing disability.

Limited research is focusing on predictive biomarkers and functional outcomes on this topic [31]. Thus, the challenge of personalizing TP in neurorehabilitation is to identify a viable way that provides omic sciences to examine multidimensional outcomes (e.g., neurobiology of injury response to treatment).

In literature, there is a growing interest about the concept of “reserve”: individual characteristics, different in each person, that could impact on the clinical manifestation and on the course of the pathology [32,33,34]. The concept is divided into a passive-quantitive model and active model [35,36]. The active model is called “cognitive reserve” and is the ability to use more flexible and efficient cognitive strategies, which can emerge from different life experiences [32]. It is not easily quantifiable and it is detected by proxies like education, hobbies, occupation, etc. ([37,38,39]. The passive-quantitative model defined as “brain reserve”, in which reserve is defined as the amount of damage that the brain can tolerate before it manifests itself clinically and can cause impairment of a function (motor/cognitive), both as a consequence of physiological processes aging and acquired and degenerative pathological processes. In a pioneering study, this model was addressed in terms of neurons and synapses arguing that better overall performance corresponds to greater brain mass direct *connected* with brain plasticity [33,36,40]. Subsequently, plasticity was considered a key feature to explain individual differences in coping with brain damage, depending on structural factors such as brain size and number of synapses [32]. The brain reserve is still considered a protective factor for many neurodegenerative diseases that can remain blocked for a long time due to the size and neural networks.

The study of brain reserve/plasticity represents the biological substrate for neurorehabilitation and an objective link between recovery and omic sciences [41].

**Figure 2:**
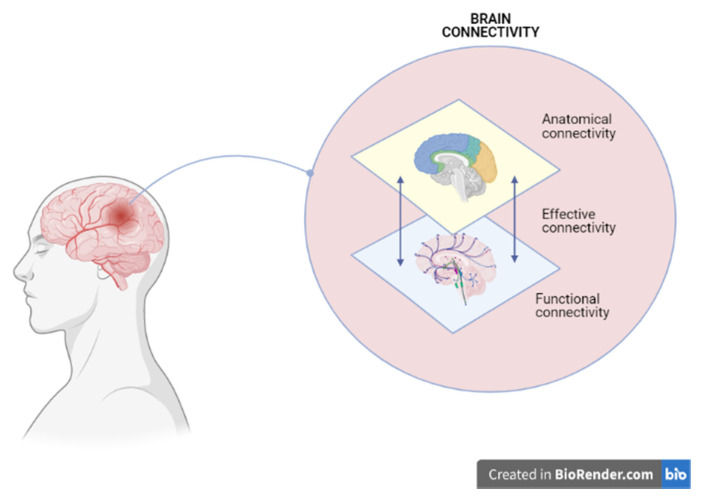
A severe brain injury may affect from one to all the patterns that constitute brain connectivity. Image created with BioRender.com (accessed on 1 March 2021).

Brain plasticity is the ability of the brain to reorganize itself at a micro- and macro-structural level in physiological or pathological conditions. It plays a key role in severe brain injury rehabilitation treatment [42], and requires the presence of hierarchically organized neural networks. For instance, brain connectivity is the functioning of brain networks and it refers to a pattern (Figure 2):

- of anatomical links: the bundles of fibers that connect brain areas with each other (“anatomical connectivity”);
- of statistical dependencies: the electrical and metabolic synchronization of brain areas that work in concert with each other, although anatomically distant (“functional connectivity”);
- of causal interactions: the hierarchy of relationships between these areas (“effective connectivity”).

Imaging techniques show the macroscopic elements of neuroplasticity [42]. However, in the last decade there is a growing attention about the mechanisms underlying the reorganization of networks are manifold and vary from the reorganization of neuronal membrane conductance (long term potentiation and long term depression), to the creation of new connections between neurons (synaptogenesis). The micro- and macro-structural connectivity is based on the deoxyribonucleic acid (DNA) of the brain cells whose modulation depends on individual structural factors and on environmental factors.

### 3.2. The Advent of “Rehabilomics”

Because rehabilitation requires an intimate understanding of how severe brain injuries impact disability, impairments, and QoL, PM represents a great opportunity for brain injury survivors to improve recovery [43]. In the perspective, we can consider the impact of omic sciences to address the most appropriate rehabilitation treatment on the basis of patients’ needs, characteristics and life experience [30,44,45,46].

The progress in medical genetics and genomics elucidated many biological processes [47]. Several polymorphism influence brain plasticity [48]. In particular, two genes and related polymorphisms appear to hold promise in this area: the brain derived neurotrophic factor (BDNF) and the Apolipoprotein E (apoE) [48,49,50]. Identifying those variants could help predicting more accurately the neurological prognosis of a patient following a severe brain injury.

Biomarkers can be useful in understanding pathology and prognosis in case of traumatic brain injuries: neurotransmitter hypofunction and persistent hypogonadism appeared to be connected cognitive deficits and worse clinical outcomes [51]. In the last decade, a new field of study aims to understand the biology, functioning, incidence, treatment, prognosis, and recovery for people with disability: the Rehabilomics.

Towards the systematic collection and study of clinical phenotypes and biomarkers, Rehabilomics can help in identifying relevant molecular or physiological fingerprints for anticipating long term outcome that can be linked to plasticity, treatment response, and natural recovery [43,52,53,54], especially in emergency situation [55]. Rehabilomics encompasses rehabilitation research that uses biomarkers in its design. In severe brain injury rehabilitation, this paradigm seems to be a promising providing an omic overlay to the scientific study of neurorehabilitation processes and multidimensional outcomes. This approach could provide novel opportunities to evaluate the neurobiology of complex injuries or chronic diseases and can be used to examine methods and treatments for personalized TP among patients.

## 4. Discussion and Conclusions

The integration of PM into primary, secondary, and tertiary prevention may benefit citizens, patients, healthcare professionals, decision making and industry, and ultimately will seek to contribute to better health and quality of life (QoL) for Europe’s citizens. Omic sciences are playing a crucial role in achieving personalization of care, however care goals should deal with cost-effectiveness of innovative tests, therapies and technologies. Cost-effectiveness analysis, in fact, can provide important insights to guide policy makers on the best policies related to PM. In the future, it is thinkable that once an individual has been genotyped, multiple polygenic scores could be generated for several diseases and subtypes of disease [9]. This scenario could enable a move towards predictive prevention where information on genetic risk, in addition to family history, may be more effective in setting up targeted screening programs. However, this would require careful consideration of interventions to be offered to individuals identified at elevated risk and demonstration of beneficial health impacts. In fact, the capacity in which polygenic scoring information is likely to be used will differ substantially for different conditions, depending on the underlying genetic architecture of the disease and the degree to which this information can assist clinical and public health practice. This means that determining whether polygenic scores will be useful requires an understanding of how they can inform specific clinical and public health pathways [56].

Taking into account that PM and prevention can only be successfully implemented when handled as a truly cross-sectoral topic, ethical, legal and social implications need to be taken into account. The increased amount of health information is raising novel issues related to privacy and discrimination. Moreover, cost, access and underrepresentation in genomic research have the troubling potential to exacerbate further already existing health disparities. It is imperative that research include different populations (ethnic minority and socially disadvantaged population categories) ensuring that scientific advances are impactful to all population groups by harnessing innovations in personalized medicine to reduce and eventually eliminate health disparities.

As many primary prevention interventions rely on behavioral changes, further research is also needed on programmes that support achieving these goals, including in younger populations.

For TP, the adoption of accurate biomarker for precise monitoring and early prediction of disease progression and the implementation between omic sciences and rehabilitation as Rehabilomics should be also encouraged To study genes involved in the mechanisms of functional recovery (as BDNF and its polymorphism) and in the growth and regeneration of the central and peripheral nervous system (as apoE) could allow the identification of patients with different rehabilitation outcome perspectives. The concept of “brain reserve” (and of the related cognitive reserve) represents a promising tool to provide a personalized treatment able to provide a more succesful recovery. A more effective rehabilitation program that reduces disability and addresses social reintegration, represents an advantage for patients, society and National Healthcare System because of the reduction of direct and indirect costs (particularly high for severe neurological and neurodegenerative pathologies) and of the reaching of a better QoL for patient and his/her relatives.

Last but not least, key factors for a successful implementation of PM are that all the stakeholders (professionals in healthcare, citizens, and patients) are engaged and committed. An investment for the literacy, education, and capacity building of healthcare professionals is needed. This comprises the inclusion of PM-related issues in the curricula of basic healthcare degrees, as well as lifelong training for medical doctors and other healthcare professionals. Simultaneously, citizens and patients are expected to adopt a range of new behaviors and practices in relation to their health care and this will be feasible only if educational, socioeconomic, ethical, and cultural hurdles are correctly addressed and managed.
